# Genetic legacy and adaptive signatures: investigating the history, diversity, and selection signatures in Rendena cattle resilient to eighteenth century rinderpest epidemics

**DOI:** 10.1186/s12711-024-00900-y

**Published:** 2024-05-02

**Authors:** Elisa Somenzi, Erika Partel, Mario Barbato, Ana María Chero Osorio, Licia Colli, Niccolò Franceschi, Roberto Mantovani, Fabio Pilla, Matteo Komjanc, Alessandro Achilli, Heidi Christine Hauffe, Paolo Ajmone Marsan

**Affiliations:** 1 DIANA Dipartimento di Scienze Animali, della Nutrizione e degli Alimenti, Università Cattolica del Sacro Cuore, Piacenza, Italy; 2 Unità risorse foraggere e produzioni zootecniche, Centro Trasferimento Tecnologico, Fondazione Edmund Mach, S. Michele all’Adige, Trento, Italy; 3 Department of Veterinary Science, Università degli Studi di Messina, Messina, Italy; 4 Dipartimento di Biologia e Biotecnologie “L. Spallanzani”, University of Pavia, Pavia, Italy; 5 Centro di Ricerca Sulla Biodiversità e sul DNA Antico, BioDNA, Università Cattolica del Sacro Cuore, Piacenza, Italy; 6 Department of Agronomy, Food, Natural Resources, Animals, and Environment, University of Padua, Padua, Italy; 7 Department of Agriculture Environment and Food Science, University of Molise, Campobasso, Italy; 8 Conservation Genomics Research Unit, Research and Innovation Centre, Fondazione Edmund Mach, S. Michele all’Adige, Trento, Italy; 9 Centro di Ricerca Nutrigenomica e Proteomica-PRONUTRIGEN, Università Cattolica del Sacro Cuore, Piacenza, Italy

## Abstract

**Background:**

Rendena is a dual-purpose cattle breed, which is primarily found in the Italian Alps and the eastern areas of the Po valley, and recognized for its longevity, fertility, disease resistance and adaptability to steep Alpine pastures. It is categorized as 'vulnerable to extinction' with only 6057 registered animals in 2022, yet no comprehensive analyses of its molecular diversity have been performed to date. The aim of this study was to analyse the origin, genetic diversity, and genomic signatures of selection in Rendena cattle using data from samples collected in 2000 and 2018, and shed light on the breed's evolution and conservation needs.

**Results:**

Genetic analysis revealed that the Rendena breed shares genetic components with various Alpine and Po valley breeds, with a marked genetic proximity to the Original Braunvieh breed, reflecting historical restocking efforts across the region. The breed shows signatures of selection related to both milk and meat production, environmental adaptation and immune response, the latter being possibly the result of multiple rinderpest epidemics that swept across the Alps in the eighteenth century. An analysis of the Rendena cattle population spanning 18 years showed an increase in the mean level of inbreeding over time, which is confirmed by the mean number of runs of homozygosity per individual, which was larger in the 2018 sample.

**Conclusions:**

The Rendena breed, while sharing a common origin with Brown Swiss, has developed distinct traits that enable it to thrive in the Alpine environment and make it highly valued by local farmers. Preserving these adaptive features is essential, not only for maintaining genetic diversity and enhancing the ability of this traditional animal husbandry to adapt to changing environments, but also for guaranteeing the resilience and sustainability of both this livestock system and the livelihoods within the Rendena valley.

**Supplementary Information:**

The online version contains supplementary material available at 10.1186/s12711-024-00900-y.

## Background

Rendena is an indigenous, dual-purpose cattle breed, which is valued by farmers for both milk and meat quality, and reared principally in the Italian Alps and the eastern areas of the Po valley. The breed is named after the Rendena valley, a UNESCO World Heritage Site nestled between the Brenta Dolomites and the Eastern Alps in the Province of Trento, Italy. This breed is relatively small, with short robust legs, small hooves and good grazing ability, and is well adapted to grazing on the steep Alpine summer pastures. Rendena cattle are also popular locally for their longevity, high fertility and disease resistance [1]. However with only 6057 animals in the national Herd Book (http://www.anare.it/) in 2022 among which 4170 are more than two-year old females, the FAO Domestic Animal Diversity Information System (https://www.fao.org/dad-is/en/) has categorized the Rendena breed as ‘vulnerable to extinction’.

Successive colonization by diverse human populations across the centuries has led to substantial human cultural diversity in the Trentino-Alto Adige/Südtirol region [2]. The Rendena valley is no exception, with a rich history of human settlements dating back to prehistoric times, according to archaeological studies [3, 4]. This historical trajectory has given rise to roughly 30 local cattle breeds across the Alpine arc [5], with the first written evidence of Rendena cattle dating to the beginning of the eighteenth century [6]. During that same period, successive epidemics of rinderpest spread throughout the Alpine cattle populations of northern Italy, including Val Rendena. To restock their herds, farmers imported cattle of Swiss origin which were phenotypically similar to the autochthonous population [6]. When the epidemics ended in the late eighteenth century, the importation of cattle ceased, and crossbreeding with neighbouring breeds was mainly limited by geographical barriers. During the nineteenth century, the Rendena breed expanded its distribution in northern Italy, especially to Veneto and Lombardy, establishing itself for a time as the most wide-spread dairy breed in the area [6]. This expansion was interrupted between the two World Wars (c. 1919–1938) when the Italian government imposed the replacement of local breeds with more productive ones. As a consequence, the number of Rendena cattle declined to a few thousand individuals [6]. In spite of such politically-motivated disincentives, the breed survived thanks to the dedication of local farmers in the Rendena valley, who continued to rear their native cattle. More recently, the Rendena breed was fully recognised and protected with the establishment of the National Association of Breeders of Rendena Cattle (A.N.A.RE) in 1981.

To date, there has been no comprehensive analyses of the molecular diversity of the Rendena breed and information on its genetic origin, molecular diversity and conservation status are still lacking. Here, we analyse a genomic single nucleotide polymorphism (SNP) dataset that includes 28 Rendena individuals sampled in 2000 and 140 individuals sampled in 2018, as well as those from other local and highly selected breeds to (i) assess changes in population genetic diversity and structure of this breed over the last two decades; (ii) establish the origin of the breed, and (iii) identify breed-specific genomic signatures of selection.

## Methods

### Sample collection, genotyping, and quality control

Fresh blood samples of 140 Rendena cattle were collected from 27 farms located in the Province of Trento, and six farms located in the Province of Padua in the region of Veneto (Italy). To maximise the representativeness of the dataset, samples were selected to minimise the number of paternal half sibs and avoid sampling within the same maternal lineage. The birth years of the sampled animals ranged from 2010 to 2017. Rendena heifers typically calve for the first time at around three years of age. These animals are maintained in the herd for four or five lactations; therefore, the average lifespan of the animals is around eight years. Blood samples were collected following the Italian and European legislation on animal welfare (D.lgs n. 146/2001, Council Directive 98/58/CE) by a licensed veterinarian, according to the European directive 2010/63 during annual screening campaigns. In addition, 28 Rendena samples from the year 2000, collected during a previous European research project (EU RESGEN CT 98–118), were included in our analysis.

Data from 17 local breeds from the Alpine region, including 24 Rendena samples collected in the region of Veneto, were available from an independent investigation on Alpine cattle biodiversity [5]. Moreover, publicly available genotype data of 11 Central European [5, 7, 8] and two unpublished East European cattle breeds were included in the dataset (see Additional file 1: Table S1).

DNA was extracted at the laboratory of Università Cattolica del Sacro Cuore (Italy) from the 168 Rendena samples using the GenElute Mammalian Genomic DNA Miniprep kit (Sigma, St Luis, MO, USA) according to the manufacturer’s instructions. DNA samples were genotyped with the GeneSeek (Neogene, Lincoln, Nebraska) GGP Bovine 100 K Illumina SNP chip (Illumina Inc.). For this study, two datasets were built: the first comprising the data from the 168 new Rendena samples genotyped with the GGP Bovine 100 K Illumina SNP chip, and the second also including data from the 30 Alpine, Central and East European breeds, after updating marker positions of all the samples to the ARS-UCD1.2 bovine reference genome [9], and retaining only consensus markers between different SNP chips. In the merged dataset, Rendena cattle from this investigation were labelled RENgen (after the Rendenagen project), and the subsets of animals sampled in years 2000 and 2018 named Rendena2000 and Rendena2018, respectively. The publicly available data from 24 extra Rendena samples were labelled REN.

Both datasets were quality-controlled using the PLINK v1.9 software [10] to achieve a SNP call rate > 0.98, an individual call rate > 0.98, and a minor allele frequency (MAF) > 0.01. Markers located on sex chromosomes or with an unknown map position were removed. Pruning for linkage disequilibrium (LD) was performed with the PLINK flag –*indep-pairwise* setting an r^2^ value of 0.2, a window of 50 and a step size of 5.

### Characterization of the Rendena breed within local and European genetic contexts

The merged dataset was used to investigate potential gene flow and admixture that may have occurred over centuries between the Rendena and other breeds. We calculated observed (H_O_) and expected (H_E_) heterozygosity for each breed included in this study using PLINK; instead, inbreeding coefficient (F_IS_) values were calculated using the Arlequin v3.5.2.2 software, with the threshold for statistical significance set at p < 0.05 [11]. To estimate the effective population size (*N*_*e*_), two different approaches were used: SneP v1.1 [12] with default parameters (to facilitate comparison with results from previous publications on local cattle breeds) and currentNe v1.0 [13]. Runs of homozygosity (ROH)-derived inbreeding coefficients (F_ROH_) were calculated independently for each population, as implemented in the R package detectRUNS v 0.9.6 [14]. ROH values were calculated with PLINK using the following parameters: (i) sliding window of 50 SNPs; (ii) a maximum of one heterozygous genotype and one missing genotype allowed per ROH; (iii) minimum number of SNPs in a ROH calculated according to the formula described by Purfield and colleagues [15]; (iv) a minimum ROH length of 500 kb; (v) a minimum density of one SNP per 100 kb and; (vii) a maximum gap of 1 Mb between consecutive homozygous SNPs.

Changes in population structure were assessed with a principal component analysis (PCA) that was performed using PLINK and plotted with the R software [16]. The software Admixture v1.3.0 implements a maximum-likelihood based approach to infer ancestry proportions by evaluating a *K* number of theoretical ancestral populations [17]. Here, we tested ancestry solutions for *K* ranging from 2 to 30, and the built-in cross-validation (CV) error approach was used to identify the best fitting value of *K*. To alleviate sample-size bias in the analysis, we used the BITE v1.2 R package [18] to subset the most numerous populations to a maximum of 30 individuals, selected to mimic the population structure of the full set (the subset of 30 Rendena samples includes animals from previously published data and from this study).

Relationships among populations were explored by pairwise Reynolds genetic distances (computed with a custom script) and visualized with a Neighbour-net graph produced with the SplitsTree v4.14.6 software [19]. To assess population splits and gene flow events, the Treemix software [20] was run on the whole dataset and on a reduced dataset comprising the following breeds: Rendena, Brown Swiss (BSW), Original Braunvieh (OBV) and Murnau-Werdenfelser (MWF), using Jersey (JER) as the root. We set the block size for jackknifing (-*k*) to 500 SNPs and tested a number of migration events (*m*) from 0 to 10 in the whole dataset and from 0 to 5 in the reduced set.

### Evaluation of the recent genetic management of the Rendena breed

We used the SNP chip data from 168 Rendena individuals to evaluate how selection has affected the genetic structure and level of inbreeding of the Rendena cattle population over the past 18 years, by comparing data from samples collected in the year 2000 with data from samples collected for this study in the year 2018. ROH were calculated independently for each of the two groups with PLINK according to the parameters given above, and used to evaluate the inbreeding level in Rendena2000 and Rendena2018, using individual and per-group F_ROH_ values calculated with the R package detectRUNS. To enhance the reliability of the comparison between populations with different sample sizes (N = 28 in Rendena2000 and N = 140 in Rendena2018), for Rendena2018, ROH mean statistics were computed following 1000 bootstrap iterations of 28 randomly selected animals. Confidence intervals (95%) were computed on the results obtained from the two Rendena populations. Changes in population structure were assessed with PCA performed with PLINK and visualised in R. To further test the genetic divergence between the two populations, a discriminant analysis of principal components (DAPC) was performed and visualised in R using the package Adegenet [21].

### Analyses of signatures of selection

ROH, cross-population extended haplotype homozygosity (XP-EHH) and integrated haplotype score (iHS) were used to scan for signatures of selection in the Rendena breed, which may have occurred at different times during the history of the breed.

#### ROH

ROH values were investigated in the RENgen sample set to detect human and environmental-mediated selection. To identify regions putatively under selection, we used the R package detectRUNS to calculate the frequency with which each SNP falls within a ROH [14]. SNPs with values in the top 1% of the distribution were identified as potential locations under selection [22]. The R package GALLO [23] was used to annotate each significant SNP, considering a flanking region of 17.8 kb, upstream and downstream of the target SNP, corresponding to half the mean distance between adjacent markers.

#### EHH-based metrics

To evaluate genome-wide selection, we used EHH and estimated the decay of haplotype homozygosity as genetic distance increases. Two EHH-based metrics were calculated, iHS within population and XP-EHH between pairs of populations using the rehh v2.0 package [24]. The first analysis was performed on the RENgen samples only, while the XP-EHH analysis was also performed on the Original Braunvieh and modern Brown Swiss breeds (BSW and BSW_IT), and results were compared to identify shared and divergent signatures of selection.

To polarise the iHS analysis, we used the ancestral and derived allele cattle data from Naji and colleagues [25]. However, as information on the ancestral alleles was not available for all markers in the dataset, we performed the analysis in two stages. First, the subset of markers with available ancestral allele information was analysed, then the analysis was repeated using the entire dataset, assuming the major allele is the ancestral allele. The significance threshold was set at a p-value = 0.01.

The XP-EHH metric identifies selective sweeps where an allele undergoes strong directional selection in one population while remaining polymorphic in the overall population [26]. We applied XP-EHH to compare the Rendena versus both the Original Braunvieh and the Modern Brown Swiss breeds. Markers with an XP-EHH score in the top 1% of the standardized distribution were identified as under putative positive selection. Only signatures of selection relative to the Rendena breed are discussed below. Markers with a value above the threshold were annotated with the GALLO R package considering a flanking region of 17.8 kb, upstream and downstream the target SNP as described above.

## Results

### Sample collection, genotyping and quality control

Following quality control of the two datasets, 75,157 SNPs and 167 out of 168 genotyped individuals were retained in the dataset that consisted exclusively of the Rendena samples from the Rendenagen project (RENgen), and 16,785 SNPs and 908 individuals for the dataset including Rendenagen samples, previously published Rendena samples and the breeds chosen for biodiversity analyses.

### Characterization of the Rendena breed within local and European genetic contexts

The values for H_O_ and H_E_ ranged from 0.30 and 0.29, respectively, for the JER breed, to 0.40 and 0.40 for the RENgen samples (Table 1). *P*-values associated with the inbreeding coefficient F_IS_ estimates were not statistically significant (significance at p < 0.05; see Additional file 2: Table S2). Genomic inbreeding estimated from ROH gave values ranging from 0.02 (Cika) to 0.12 (Jersey), with RENgen having values of 0.06 and 0.09 for the Rendena2000 and Rendena2018 populations, respectively. Contemporary *N*_*e*_ estimated by the SNeP software were 180 and 237 for the RENgen populations sampled in the years 2000 and 2018, respectively (Table 1). *N*_*e*_ values of 180 and 269 were obtained from currentNe for the Rendena2000 and Rendena2018 populations, respectively, with 90% confidence intervals ranging from 154 to 209 for the Rendena2000 population, and from 236 to 284 for the Rendena2018 population (see Additional file 3: Table S3).

**Table 1:** Dataset description including breed name, acronym, country of origin, number of samples (N) in the working dataset, observed (H_O_) and expected (H_E_) heterozygosity with standard deviation (SD), genomic inbreeding (F_ROH_) and contemporary values of *N*_*e*_ computed with SNeP (S-*Ne*)

Breed name	Acronym	Country	N	H_O_ ± SD	H_E_ ± SD	F_ROH_	S-*N*_*e*_
Abondance	ABO	France	20	0.35 ± 0.19	0.32 ± 0.16	0.02	65
Barà Pustertaler	BPU	Italy	24	0.35 ± 0.17	0.34 ± 0.14	0.04	91
Blonde d'Aquitaine	BLO	France	5	0.36 ± 0.25		–	
Braunvieh	BRV	Switzerland	30	0.32 ± 0.18	0.31 ± 0.16	0.09	75
Brown Swiss	BSW	Switzerland	19	0.32 ± 0.19	0.31 ± 0.17	0.09	52
Bulgarian grey	BGR	Bulgaria	20	0.39 ± 0.17	0.35 ± 0.13	0.11	41
Burlina	BUR	Italy	24	0.35 ± 0.17	0.34 ± 0.15	0.04	83
Charolais	CHA	France	20	0.37 ± 0.16	0.36 ± 0.13	0.03	95
Chianina	CHI	Italy	16	0.33 ± 0.21	0.32 ± 0.17	0.05	53
Cika	CIK	Slovenia	26	0.36 ± 0.16	0.35 ± 0.14	0.02	104
Fleckvieh	FLV	Austria	30	0.34 ± 0.17	0.33 ± 0.15	0.03	122
Guernsey	GNS	France	21	0.32 ± 0.18	0.32 ± 0.16	0.10	63
Holstein	HOL	Netherlands	32	0.34 ± 0.17	0.34 ± 0.16	0.09	70
Hungarian grey	HUN	Hungary	29	0.38 ± 0.15	0.37 ± 0.12	0.08	71
Italian Brown	BSW_IT	Italy	32	0.31 ± 0.18	0.30 ± 0.17	0.11	61
Italian Simmental	SIM	Italy	31	0.34 ± 0.17	0.33 ± 0.15	0.03	108
Jersey	JER	United Kingdom	19	0.30 ± 0.20	0.29 ± 0.18	0.12	48
Limousin	LMS	France	44	0.34 ± 0.16	0.33 ± 0.15	0.03	156
Montbeliard	MON	France	20	0.34 ± 0.19	0.32 ± 0.16	0.05	56
Murnau-Werdenfelser	MWF	Germany	30	0.35 ± 0.18	0.33 ± 0.16	0.03	71
Original Braunvieh	OBV	Switzerland	35	0.34 ± 0.17	0.33 ± 0.15	0.04	121
Pezzata Rossa d’Oropa	PRO	Italy	23	0.33 ± 0.17	0.33 ± 0.16	0.05	78
Piedmontese	PMT	Italy	24	0.35 ± 0.17	0.34 ± 0.15	0.01	113
Pinzgauer	PIN	Austria	24	0.35 ± 0.17	0.34 ± 0.15	0.05	73
Pustertaler	PUS	Italy	24	0.34 ± 0.18	0.32 ± 0.16	0.05	60
Rendena	REN	Italy	24	0.33 ± 0.18	0.33 ± 0.16	0.06	77
Rendena*	RENgen (Rendena2000)	Italy	28	0.41 ± 0.14	0.4 ± 0.12	0.06	180
Rendena*	RENgen (Rendena2018)	Italy	139	0.40 ± 0.12	0.39 ± 0.12	0.09	237
Romagnola	RMG	Italy	24	0.32 ± 0.19	0.31 ± 0.17	0.08	70
Tarine	TAR	France	18	0.336 ± 0.19	0.32 ± 0.16	0.03	70
Varzese Ottonese	VAR	Italy	30	0.35 ± 0.17	0.34 ± 0.15	0.05	85
Vosgienne	VOS	France	20	0.35 ± 0.18	0.33 ± 0.15	0.02	73

FROH and *N*_*e*_ were computed for breeds with a sample size larger than 10

^*^Current study

**Fig. 1:**
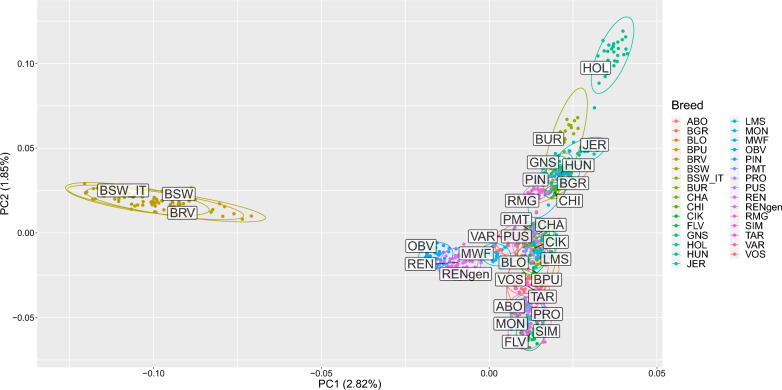
Principal component analysis: PC1 vs. PC2. The percentage values within brackets refer to the proportion of variance explained by the displayed principal components. See Table 1 for breed acronyms

In the PCA, the first and second principal components (PC) explained 2.82 and 1.85% of the total variance, respectively. The first PC discriminated the modern Brown Swiss group of breeds (BSW_IT, BSW, BRV) from all the other breeds included in the analysis (Fig. 1). In the first PC, Rendena clusters with the Original Braunvieh and Murnau-Werdenfelser Alpine breeds, but forms a separate group from the modern Brown Swiss cluster. The second PC separates two groups, one including the Alpine breeds, positioned according to a west-to-east geographic gradient, and a second comprising Podolian breeds. Burlina falls within this latter group, because of its proximity with Holstein, located at the upper extreme of the second PC (Fig. 1, and see Additional file 4: Fig. S1).

**Fig. 2:**
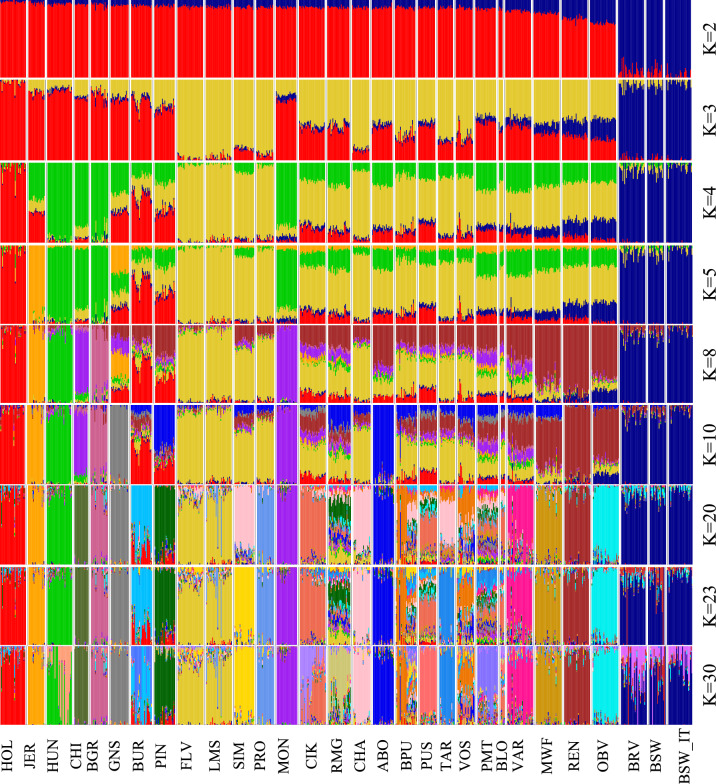
Admixture analysis. Admixture plot including clustering solutions from 2 to 10, 20, 23 and 30. Populations are ordered according to *K* = 2 values. See Table 1 for breed acronyms

ADMIXTURE analysis at *K* = 2 separates the modern Brown Swiss breeds from the other breeds, with Original Braunvieh and Rendena sharing more of the Brown Swiss-like ancestral component than the rest of the populations in the dataset (Fig. 2). *K* = 3 showed a genetic component shared by all the alpine breeds, but it was more markedly present in breeds from the western area of the Alps (FLV, SIM, PRO, MON, ABO).

As illustrated in Fig. 2, at *K* = 8 an ancestry component (brown colour) was detected, primarily shared by Rendena, Original Braunvieh and Murnau-Werdenfelser, but also present in all the alpine breeds as well as in the Varzese breed from the north-western part of the Apennine region. All *K* values greater than 8 progressively distinguished individual breeds (Fig. 2, see Additional file 5: Fig. S2). The lowest CV error value corresponded to *K* = 23 (see Additional file 6: Fig. S3) at which all breeds had distinguishable patterns, with the exception of the modern Brown Swiss group, the Fleckvieh and Simmental, and the REN and RENgen samples.

**Fig. 3:**
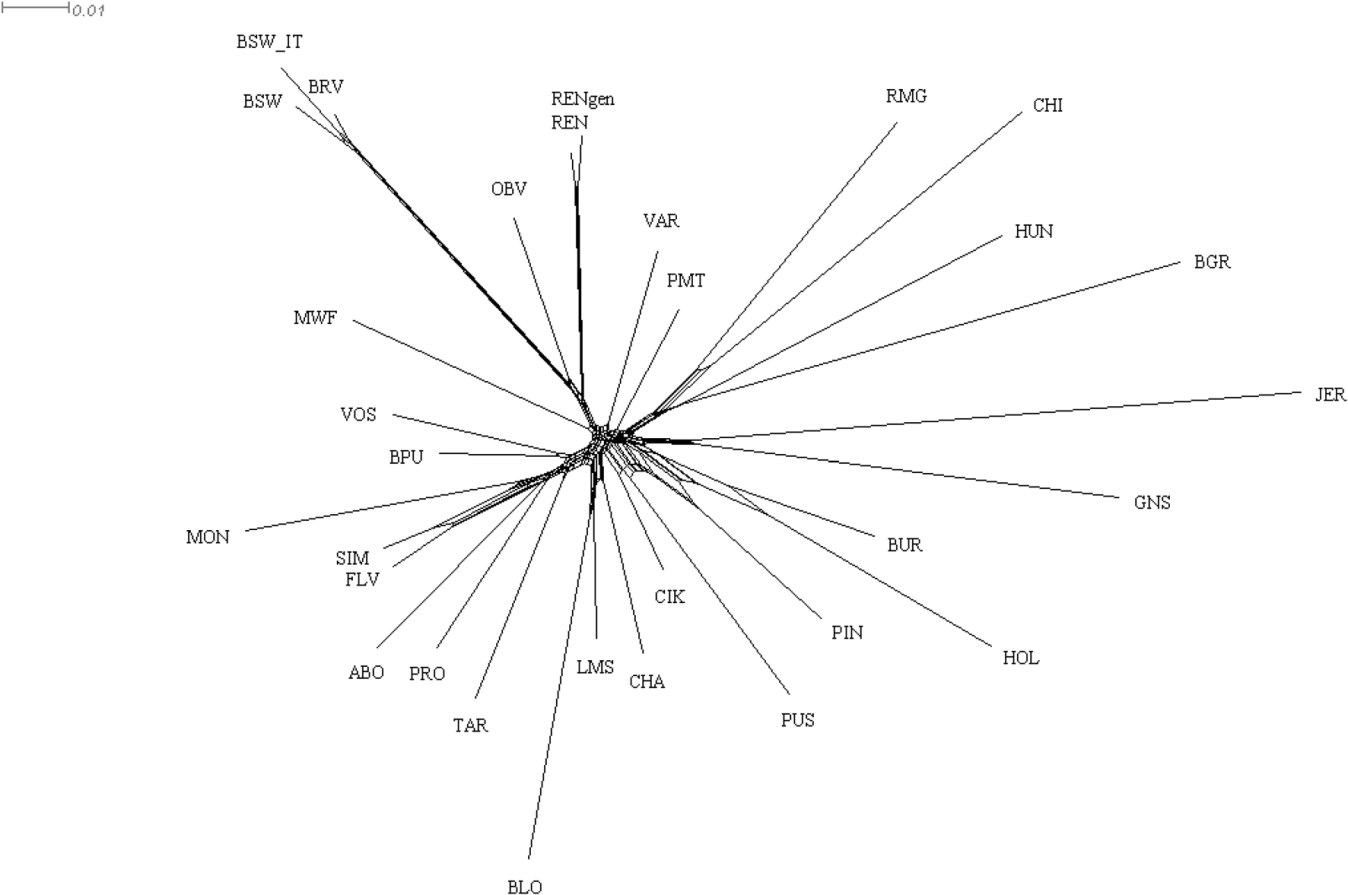
Neighbour-network reconstruction based on Reynolds’ genetic distances between breeds. Distance scale is represented in the upper left side of the figure. See Table 1 for breed acronyms

The Neighbour-net graph based on Reynold’s genetic distances showed that the Rendena breed has an ancestral relationship with the Brown Swiss cluster (Fig. 3). However, as seen in the PCA, the Rendena breed appears to be closer to the root of the branch and to the Original Braunvieh than to the modern Brown Swiss population. The Murnau-Werdenfelser and the Varzese breeds were located nearby, confirming the genetic pattern shown by the Admixture analysis (Fig. 2).

**Fig. 4:**
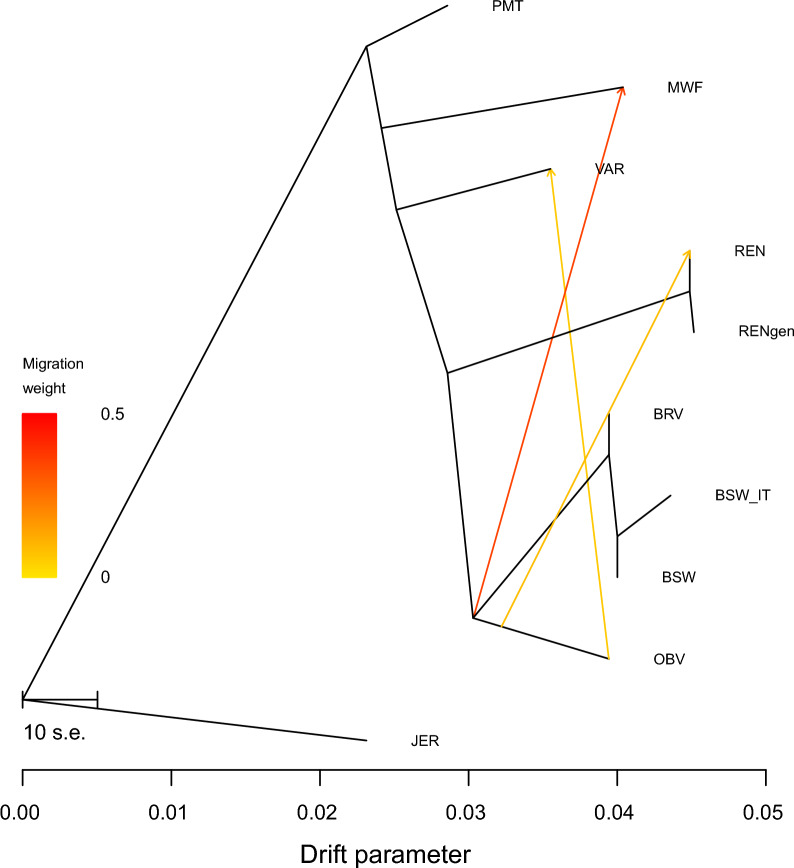
Treemix plot for *m* = 3. Migration edges between populations are represented by arrows pointing to the receiving population and coloured according to the migration weight. See Table 1 for breed acronyms

The Treemix analysis performed on the whole dataset for *m* from 2 to 10 did not identify gene flow events involving the Rendena breed. Consequently, focussed Treemix analysis was performed on a subset of populations including Rendena and the breeds that were identified by the Neighbour-net analysis as genetically closer to Rendena (i.e., BSW, BRV, BSW_IT, OBV, MWF, VAR and PMT), using Jersey as outgroup. The analysis identified gene flow events from Original Braunvieh towards the Rendena, Varzese and the Murnau-Werdenfelser (Fig. 4).

### Evaluation of recent genetic management of the Rendena breed

**Fig. 5:**
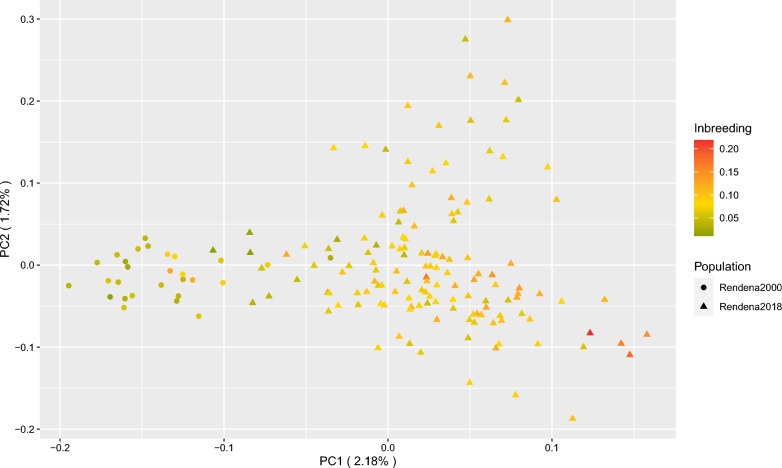
Principal component analysis (PC1 vs. PC2) of the Rendena population sampled in 2000 (circles) and 2018 (triangles). Colour gradient represents the inbreeding level calculated as F_ROH_ for each individual. The percentages of variance explained by each principal component are given in brackets

Data from Rendena cattle sampled in the year 2000 (Rendena2000, n = 28) and year 2018 (Rendena2018, n = 140) were analysed independently to assess changes in population structure and molecular diversity over the past ~ 20 years. H_O_ and H_E_ values calculated for the two populations were almost identical, respectively 0.41 ± 0.14 and 0.4 ± 0.11 for the Rendena2000, and 0.40 ± 0.13 and 0.39 ± 0.12 for Rendena2018. In contrast, the mean F_ROH_ of Rendena2000 (0.06) was significantly lower than that of Rendena2018 (0.09) (p < 0.001) (Table 2). A mean number of ROH per individual of 27.68 for Rendena2000 and 30.15 for Rendena2018 (p < 0.001; Table 2) was recorded. The average ROH length was 5.21 Mb and 7.01 Mb for Rendena2000 and Rendena2018, respectively (p < 0.001; Table 2). Rendena2000 had a higher frequency of short ROH segments, between 0–2 Mb and 2–4 Mb, while Rendena2018 showed a higher occurrence of medium to long segments, with almost 20% of the ROH segments being longer than 16 Mb (Table 2). PCA identified two partially overlapping clusters (Fig. 5), also confirmed by DAPC analysis (see Additional file 7: Fig. S4).

**Table 2:** Summary statistics of ROH for the two Rendena populations sampled in 2000 and 2018

	Mean number of ROH per individual (lower – upper 95% CI)	Mean ROH length per individual Mb (lower – upper 95% CI)	ROH frequency distribution per length class				F_ROH_ (lower – upper 95% CI)
			0–2 Mb	2–4 Mb	4–8 Mb	> 16 Mb	
Rendena 2000	27.68 (24.91–30.44)	5.21 (4.55–5.63)	0.21	0.39	0.25	0.04	0.058 (0.048–0.068)
Rendena 2018	30.15 (30.05–30.26)	7.01 (6.99–7.04)	0.05	0.34	0.33	0.19	0.087 (0.086–0.087)

*CI* confidence interval

### Analyses of signatures of selection

#### ROH

**Fig. 6:**
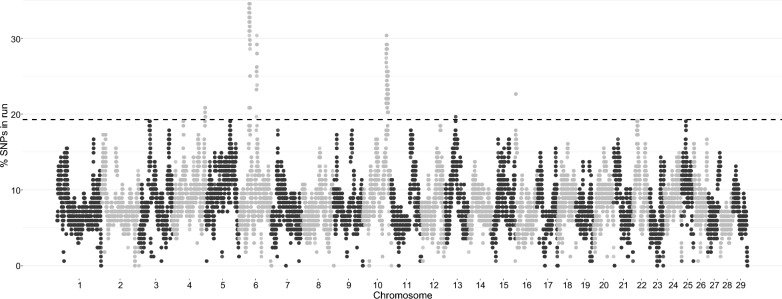
Manhattan plot representing the proportion of times each SNP falls within a ROH in the RENgen population. The dashed line represents the significance threshold

Signatures of selection were investigated in RENgen dataset assessing the proportion of times each SNP falls inside an ROH (Fig. 6). This analysis led to the identification of 769 candidate SNPs. These SNPs were subsequently annotated, resulting in a total of 114 genes located on ten chromosomes (BTA3, 4, 5, 6, 10, 12, 13, 16, 22, and 25; see Additional file 8: Table S4). The most represented chromosome was BTA10, on which 48 annotated genes were identified, followed by BTA6 and BTA22 with 19 and 17 genes, respectively.

#### EHH-based metrics

iHS analysis of the Rendena cattle was initially performed on a dataset of 167 animals and only on the 7626 SNPs for which the ancestral allele information was available. A threshold of significance was set to a nominal p-value of 0.01 genome-wide; however, no SNP was significant. The analysis was then performed on the whole set of SNPs, considering the major allele as ancestral (see Additional file 9: Fig. S5). In this case, two SNPs on BTA7 were identified as being under selection, although no gene was annotated in the 17.8 kb region upstream and downstream of these loci.

**Fig. 7:**
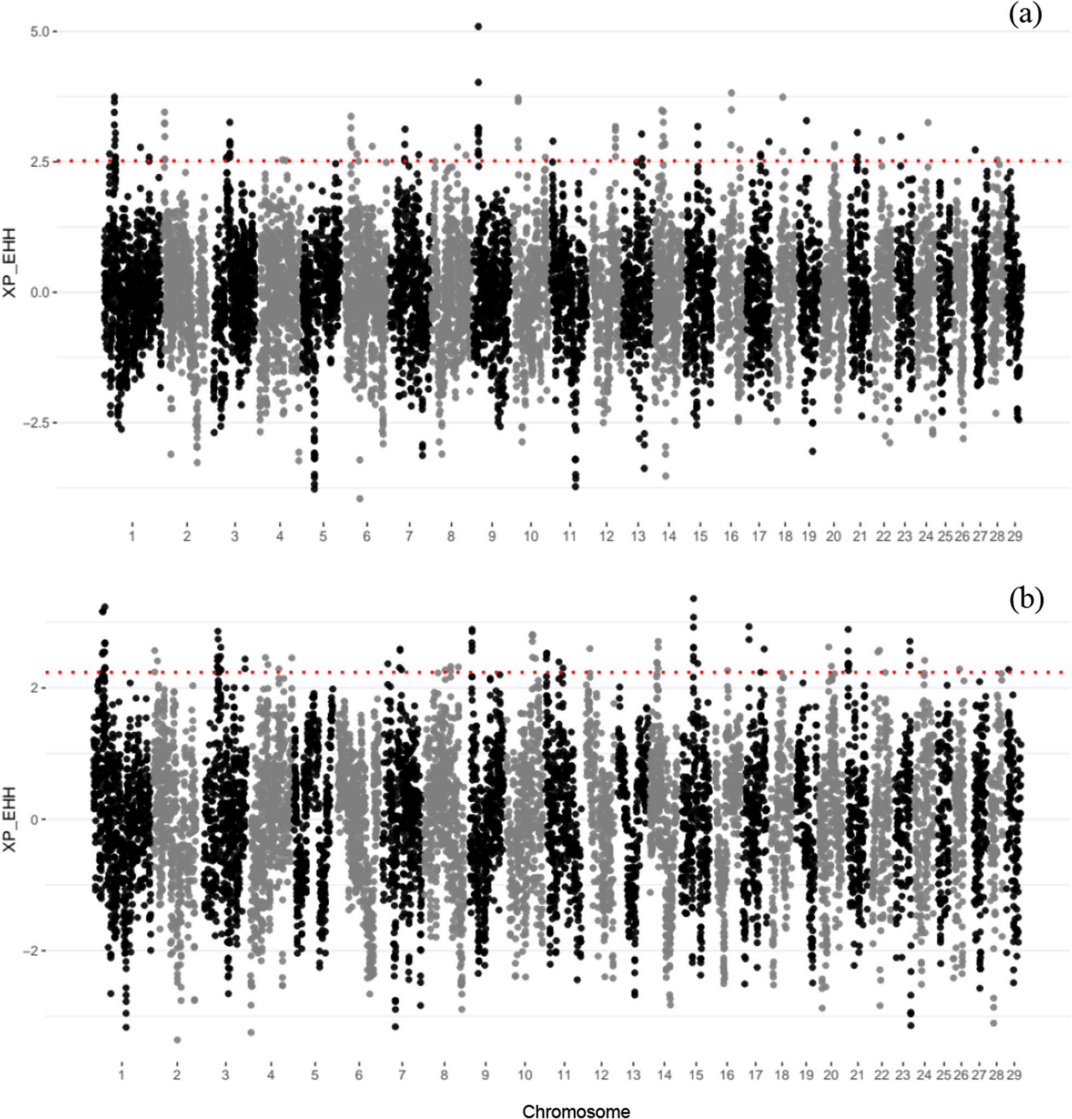
XP-EHH analysis of **a** REN vs OBV and **b** REN vs Modern Brown Swiss (BSW + BSW_IT). The significance threshold is represented by the red dotted line

XP-EHH analysis was used to compare EHH profiles of the Rendena breed with the Original Braunvieh and the modern Brown Swiss breeds, to detect signatures indicating divergent selection (Fig. 7). In the comparison between Rendena and Original Braunvieh, 22 SNPs with values falling within the top 1% of the standardized distribution (equivalent to an XP-EHH value equal or greater than 2.52) were annotated, including SNPs on chromosomes BTA1, 2, 3, 8, 9, 14, 15, 16, and 20. In addition, in the comparison between Rendena and Modern Brown Swiss, 35 SNPs exceeded the threshold of 2.23, corresponding to the top 1% of the standardized distribution, and were distributed across 13 chromosomes (BTA1, 2, 3, 7, 8, 9, 11, 14, 15, 16, 17, 18, and 23). Among identified markers, 17 SNPs on eight different chromosomes scored a XP-EHH value in the top 1% of the standardized distribution in both comparisons (see Additional file 10: Table S5). In the 17.8 kb region upstream and downstream of the identified SNPs, ten genes were annotated in the comparison between Rendena and Original Braunvieh and 12 in the comparison with Modern Brown Swiss. Seven of the annotated genes were in common between the two comparisons (see Additional file 10: Table S5). There was no shared signature of selection between the two methods.

## Discussion

The documented history of Rendena cattle can be traced back to the eighteenth century when restocking efforts were initiated following a series of devastating rinderpest epidemics, which required the introduction of cattle from neighbouring Alpine valleys in different countries [6]. Subsequently, between 1920 and 1940, national legislation mandated the replacement of local cattle with more productive breeds. However, in the Rendena valley, livestock farmers strongly opposed and ultimately resisted this mandate, leading to an exemption from the law in 1937 [6].

Consistent with these historical documents, our admixture analysis at *K* = 8 (Fig. 2), revealed a genetic component shared among a number of breeds from the Alps and the Po valley. This result is further supported by the Treemix results (Fig. 4), which indicate three gene flow events affecting the northern Italian cattle. The source population was always the Original Braunvieh with three receiving breeds: the Varzese from the western Po valley, the Murnau-Werdenfelser and the Rendena. These genetic signatures likely reflect historical restocking efforts across the Alps, extending down into the Po valley, areas significantly affected by rinderpest [27].

The PCA and Neighbour-net analyses underscored the genetic proximity of the Rendena breed to the Original Braunvieh, a breed with roots tracing back to mediaeval Switzerland and subsequently exported to western Austria and the Italian (southern) Alps in the fourteenth century. In addition, the Rendena breed shows genetic affinity to the highly endangered Murnau-Werdenfelser breed, which originated from crossbreeding between the local Bavarian breeds Oberinntaler and Braunvieh (Figs. 1, 2, and 4) [28]. These findings align with historical records indicating that, in the eighteenth century, the Rendena valley was primarily restocked with Swiss-origin cattle that shared similar morphological traits and suitability to mountainous environments. However, in the twentieth century, the breed also experienced an introgression from the transboundary Brown Swiss population imposed by national legislation, making it challenging to distinguish the genetic signatures of these two events.

Our comparative analysis of Rendena animals sampled at an 18-year interval (2000 vs 2018) revealed a notable mean inbreeding level in 2018. This increase was corroborated by the comparison of the mean number of ROH per individual, which was significantly larger in the 2018 sample (Table 2). In addition, the mean length of ROH segments in the 2018 sample exceeded that recorded in 2000, underscoring a recent escalation in inbreeding. However, this level of inbreeding remains comparable to values recorded for other local breeds from the Alps. Notably, *N*_*e*_ was larger for the 2018 sample, which is potentially due to a sampling strategy tailored to select only female samples from diverse maternal lineages and this may not have been the case for the 2000 samples, although a change in demographic dynamics cannot be excluded. The breeding strategy currently used for the Rendena breed is designed to preserve the dual-purpose nature of the breed; however, there is more emphasis on milk characteristics, with dairy accounting for 65% of the total value of the selection index, and the remaining 35% for beef traits (http://www.anare.it/) [29]. In fact, signatures of selection, revealed by the ROH and XP-EHH analyses that compare the Rendena population with the Original Braunvieh and Brown Swiss breeds, identified distinct signatures of selection in genes related to both meat and milk production (Figs. 6 and 7). Interestingly, a signature of selection on chromosome 6, identified in the ROH analysis, encompasses several genes that are linked to milk production, such as *ABCG2* [30–32], *HERC3* [33, 34], and *HERC6* [35, 36]. In addition, it includes genes associated with meat-related traits, such as *FAM184B* [37–40] and *NCAPG* [40–42]. The *LAP3* gene, which plays a role in hormone level regulation and protein maturation, has previously been associated with body conformation and carcass traits, growth, calving ease and milk production traits in cattle [41, 43–47, 49]. Chromosomes 3 and 5 also displayed signatures of selection in genes previously associated with milk production (*RNPC3* and *ST8SIA1*) [48, 49]. The *VPS13D* gene, identified through XP-EHH analysis, is involved in cholesterol regulation and lactase persistence in humans [50, 51]. Furthermore, a homozygous segment on chromosome 5 was shared among multiple individuals and contained genes related to feed efficiency (*C2CD5*) [52] and skeletal muscle development (*PYROXD1*) [53]. The *CADM2* gene on chromosome 1 and the *SOX6* gene on chromosome 10, which were identified in a signature of selection through XP-EHH analysis, have previously been linked to growth traits and fatty acid composition in multiple studies [54–59]. The change in the selection index, which took place in 2003 by incorporating muscularity and mammary traits, has not led to discernible genetic changes between 2000 and 2018, possibly due to the brief interval between the two sampling periods, but also to the lower weighting of these traits in the selection index as compared to milk yield.

Additional signatures of selection were identified near genes associated with the immune system, in particular *TNFRSF1B* on chromosome 16, which is known for its involvement in immune response and was previously observed to be under selection in Russian native cattle breeds [60]. In addition, the *HIVEP1* gene, identified through XP-EHH analysis between Rendena and modern Brown Swiss, has been linked to susceptibility to paratuberculosis infection in Chinese Holstein and US Jersey cattle [61–63] and may also play a role in trypanotolerance in Zebu Crossbred cattle in Burkina Faso [64]. Moreover, a consistently homozygous region on chromosome 22 encompassed eight genes involved in adaptive immune responses (Fig. 6; see Additional file 8: Table S4) [65]. The large number of immune response genes under selection may be the result of the Rendena having survived a series of historical epidemics that affected the Alps over several centuries. This hypothesis finds a further confirmation in the signatures of selection related to disease resistance and immunity that were found in other breeds from the Alpine arc [58, 66, 67].

Interestingly, a signature of selection was also identified in a region on chromosome 10 that harbours six genes associated with heat tolerance, lipid and energy metabolism, and adaptation to high-altitude and cold environments [68–71] (Fig. 6). Lastly, the *RCL1* gene on chromosome 8 was also found to be under positive selection in another Alpine breed, the Valdostana Black Pied [58]. These findings are particularly notable because Rendena has been bred for centuries to thrive in Alpine valleys, where animals are typically kept outdoors during the summer months. These animals graze on steep pastures at altitudes that can, in some cases, exceed 2000 m above mean sea level, often facing extreme weather conditions which could potentially impact their productivity and health.

## Conclusions

Although sharing a common origin with breeds descending from the Brown Swiss group, Rendena has retained distinctive genetic characteristics. For example, disease resistance and adaptation to local food sources and climatic conditions appear to have evolved in response to environmental challenges, and were preserved thanks to the dedication of local breeders. Retention of these traits is fundamental to maintaining this reservoir of genetic diversity, to maximize the capacity of this traditional livestock system to effectively adapt to a changing environment. Genetic signatures of selection in contemporary Rendena are likely a consequence of human pressure to improve productivity and extrinsic challenges, such as pathogens. With more dense marker data or whole-genome sequences, future research could further explore the functional significance of the genes associated with immune response and environmental adaptation in Rendena cattle, to better elucidate mechanisms underlying disease resistance and climate resilience. Investigating the genomic basis of local adaptation may inform targeted breeding strategies that are aimed at enhancing the sustainability and productivity of indigenous breeds in the face of changing and challenging conditions. In addition, comparing data from the same population sampled over time proved a valuable tool for assessing demographic trajectories and monitoring the risk of an increase in inbreeding, since local breeds that are characterized by small population sizes and regional distributions, are particularly vulnerable to increases in inbreeding and the loss of specific adaptive features. In this context, molecular data is of fundamental importance for planning breeding strategies to maintain diversity in the population, while selecting for production and adaptative traits. Moreover, locally-adapted breeds such as the Rendena breed, represent substantial social and economic value to local communities: this breed is not only a source of cultural legacy and tradition, but also plays a role in sustaining the livelihoods of the human population, contributing to the local economy not only through dairy and meat production, but also through tourism. Given the distinctive genetic heritage of the Rendena breed and its adaptation to the Alpine environment, developed over centuries of human selection, along with its substantial social, economic, and cultural importance, preserving this breed is essential for ensuring the resilience and sustainability of both the livestock system and the economy of the Rendena valley.

### Supplementary Information

**Additional file 1:Table S1.** Breed acronyms, extended breed names, SNP chip used and data source.

**Additional file 2: Table S2.** Breed acronyms, F_IS_ values and associated *p-value.*

**Additional file 3: Table S3.** Contemporary *N*_*e*_ estimates computed with the currentNe software. The table reports breed acronyms, number of samples (N samples) in the working dataset, contemporary *N*_*e*_ computed with currentNe (S-N_e_), lower bounds of 90% confidence interval (CI lower), upper bounds of 90% confidence interval (CI upper).

**Additional file 4: Figure S1.** Principal component analysis (PC1 vs. PC3). The variance explained by each component is given as percentage in brackets. See Additional file 1: Table S1 for breed abbreviations.

**Additional file 5: Figure S2. **Circular plot of Admixture results for *K* from 2 to 30 displayed using a colour-blind friendly palette. Breeds are ordered accordi g to *K* = 2 values. See Additional file 1: Table S1 for breed abbreviations.

**Additional file 6: Figure S3.** Cross-validation error values for *K* values from 2 to 30.

**Additional file 7: Figure S4.** Discriminant analysis of principal component**.** Discriminant function 1 computed for the Rendena2000 and Rendena2018 populations.

**Additional file 8: Table S4.** For each bovine chromosome, are listed the genes annotated for the most represented SNPs in the ROH analysis for the RENgen individuals.

**Additional file 9: Figure S5.** iHS results for the Rendena breed. In grey the analysis performed with all SNPs, in red the analysis performed using only SNPs for which the information on the ancestral allele was available. The dotted red and grey lines represent the significance threshold for iHS with and without ancestral allele information, respectively.

**Additional file 10: Table S5.** Chromosome, marker, genomic position, XP-EHH values in the top 1% scored in the comparison between RENgen and Original Braunvieh data, RENgen and Brown Swiss cluster and annotated genes. In bold are markers scoring a value of XP-EHH above the threshold in both comparisons.

## Data Availability

The datasets used during the current study are available from the corresponding author on reasonable request.
