# Supplementary material for: Genetic legacy and adaptive signatures: investigating the history, diversity, and selection signatures in Rendena cattle resilient to eighteenth century rinderpest epidemics
Source: Genet Sel Evol. 2024 May 2;56:32. doi: 10.1186/s12711-024-00900-y (PMC11064358; doi:10.1186/s12711-024-00900-y)
Supplement: Supplementary file 1 — Additional file 1:Table S1. Breed acronyms, extended breed names, SNP chip used and data source. [file 12711_2024_900_MOESM1_ESM.docx]

| **Breed Acronym** | **Breed name** | **SNP chip** | **Source** |
| --- | --- | --- | --- |
| ABO | Abondance | Illumina Bovine SNP50K BeadChip | [5] |
| BPU | Barà Pustertaler | Illumina Bovine SNP50K BeadChip | [5] |
| BLO | Blonde d’Aquitaine | BovineHD Genotyping BeadChip | [8] |
| BRV | Braunvieh | Illumina Bovine SNP50K BeadChip | [5] |
| BSW | Brown Swiss | Illumina Bovine SNP50K BeadChip | [5] |
| BGR | Bulgarian Grey | BovineHD Genotyping BeadChip | This study |
| BUR | Burlina | Illumina Bovine SNP50K BeadChip | [5] |
| CHA | Charolais | Illumina BovineSNP50 | [8] |
| CHI | Chianina | BovineHD Genotyping BeadChip | [7] |
| CIK | Cika | Illumina Bovine SNP50K BeadChip | [5] |
| FLV | Fleckvieh | BovineHD Genotyping BeadChip | [7] |
| GNS | Guernsey | Illumina Bovine SNP50K BeadChip | [8] |
| HOL | Holstein | BovineHD Genotyping BeadChip | [7] |
| HUN | Hungarian Grey | BovineHD Genotyping BeadChip | This study |
| BSW_IT | Italian Brown Swiss | Illumina Bovine SNP50K BeadChip | [5] |
| JER | Jersey | Illumina Bovine SNP50K BeadChip | [5] |
| LMS | Limousin | Illumina Bovine SNP50K BeadChip | [8] |
| MWF | Marnau-Werdenfelser | Illumina Bovine SNP50K BeadChip | [5] |
| MON | Montbeliard | Illumina Bovine SNP50K BeadChip | [5] |
| OBV | Original Braunvieh | Illumina Bovine SNP50K BeadChip | [5] |
| PRO | Pezzata Rossa d’Oropa | Illumina Bovine SNP50K BeadChip | [5] |
| PMT | Piemontese | BovineHD Genotyping BeadChip | [7] |
| PIN | Pinzgauer | Illumina Bovine SNP50K BeadChip | [5] |
| PUS | Pustertaler | Illumina Bovine SNP50K BeadChip | [5] |
| RENgen (Rendena2000 and Rendena2018) | Rendena | GGP Bovine 100K Illumina SNP chip | This study |
| REN | Rendena | Illumina Bovine SNP50K BeadChip | [5] |
| RMG | Romagnola | BovineHD Genotyping BeadChip | [7] |
| SIM | Simmental | Illumina Bovine SNP50K BeadChip | [5] |
| TAR | Tarine | Illumina Bovine SNP50K BeadChip | [5] |
| VAR | Varzese Ottonese | Illumina Bovine SNP50K BeadChip | [5] |
| VOS | Vosgienne | Illumina Bovine SNP50K BeadChip | [5] |
