# Supplementary material for: Genetic legacy and adaptive signatures: investigating the history, diversity, and selection signatures in Rendena cattle resilient to eighteenth century rinderpest epidemics
Source: Genet Sel Evol. 2024 May 2;56:32. doi: 10.1186/s12711-024-00900-y (PMC11064358; doi:10.1186/s12711-024-00900-y)
Supplement: Supplementary file 2 — Additional file 2: Table S2. Breed acronyms, FIS values and associated p-value. [file 12711_2024_900_MOESM2_ESM.docx]

| Breed Acronym | F_IS_ value | P-value |
| --- | --- | --- |
| ABO | -0.04672 | 0.838710 |
| BGR | -0.05876 | 0.865103 |
| BLO | 0.00211 | 0.423265 |
| BPU | -0.00094 | 0.504399 |
| BRV | -0.01267 | 0.635386 |
| BSW | -0.01687 | 0.593353 |
| BSW_IT | 0.01248 | 0.624633 |
| BUR | -0.00640 | 0.553275 |
| CHA | -0.00337 | 0.588465 |
| CHI | -0.01157 | 0.640274 |
| CIK | -0.00705 | 0.533724 |
| FLV | -0.00571 | 0.515152 |
| GNS | 0.02025 | 0.363636 |
| HOL | -0.00953 | 0.568915 |
| HUN | -0.02813 | 0.720430 |
| JER | -0.01127 | 0.582600 |
| LMS | 0.00309 | 0.461388 |
| MON | -0.04346 | 0.797654 |
| MWF | -0.04872 | 0.867058 |
| OBV | -0.00599 | 0.552297 |
| PIN | -0.01871 | 0.644184 |
| PMT | -0.01028 | 0.621701 |
| PRO | 0.00105 | 0.477028 |
| PUS | -0.03539 | 0.778104 |
| REN | -0.00015 | 0.535679 |
| RENgen | -0.01377 | 0.676442 |
| RMG | -0.00468 | 0.563050 |
| SIM | -0.01096 | 0.590420 |
| TAR | -0.01300 | 0.601173 |
| VAR | -0.00344 | 0.565982 |
| VOS | -0.02202 | 0.636364 |
