# Supplementary material for: Genetic legacy and adaptive signatures: investigating the history, diversity, and selection signatures in Rendena cattle resilient to eighteenth century rinderpest epidemics
Source: Genet Sel Evol. 2024 May 2;56:32. doi: 10.1186/s12711-024-00900-y (PMC11064358; doi:10.1186/s12711-024-00900-y)
Supplement: Supplementary file 3 — Additional file 3: Table S3. Contemporary Ne estimates computed with the currentNe software. The table reports breed acronyms, number of samples (N samples) in the working dataset, contemporary Ne computed with currentNe (S-Ne), lower bounds of 90% confidence interval (CI lower), upper bounds of 90% confidence interval (CI upper). [file 12711_2024_900_MOESM3_ESM.docx]

| Acronym | N samples | C-Ne | CI lower | CI upper |
| --- | --- | --- | --- | --- |
| ABO | 20 | 115 | 73 | 181 |
| BPU | 24 | 38 | 30 | 49 |
| BLO | 5 |  |  |  |
| BRV | 30 | 307 | 192 | 492 |
| BSW | 19 | 87 | 57 | 134 |
| BGR | 20 | 24 | 18 | 30 |
| BUR | 24 | 48 | 37 | 64 |
| CHA | 20 | 890 | 329 | 2406 |
| CHI | 16 | 146 | 80 | 269 |
| CIK | 26 | 61 | 46 | 81 |
| FLV | 30 | 444 | 258 | 763 |
| GNS | 21 | 31 | 24 | 40 |
| HOL | 32 | 47 | 38 | 59 |
| HUN | 29 | 48 | 38 | 61 |
| BSW_IT | 32 | 52 | 42 | 65 |
| SIM | 31 | 87 | 66 | 115 |
| JER | 19 | 62 | 43 | 90 |
| LMS | 44 | 78 | 64 | 94 |
| MON | 20 | 60 | 42 | 86 |
| MWF | 30 | 70 | 54 | 91 |
| OBV | 35 | 388 | 247 | 610 |
| PRO | 23 | 36 | 28 | 46 |
| PMT | 24 | 65 | 48 | 89 |
| PIN | 24 | 56 | 42 | 75 |
| PUS | 24 | 34 | 27 | 43 |
| REN | 24 | 639 | 469 | 870 |
| RENgen (Rendena2000) | 28 | 180 | 154 | 209 |
| RENgen (Rendena2018) | 139 | 269 | 236 | 284 |
| RMG | 24 | 60 | 44 | 81 |
| TAR | 18 | 49 | 34 | 71 |
| VAR | 30 | 34 | 28 | 41 |
| VOS | 20 | 26 | 20 | 34 |
