# Supplementary figures and images for: Genetic legacy and adaptive signatures: investigating the history, diversity, and selection signatures in Rendena cattle resilient to eighteenth century rinderpest epidemics
Source: Genet Sel Evol. 2024 May 2;56:32. doi: 10.1186/s12711-024-00900-y (PMC11064358; doi:10.1186/s12711-024-00900-y)

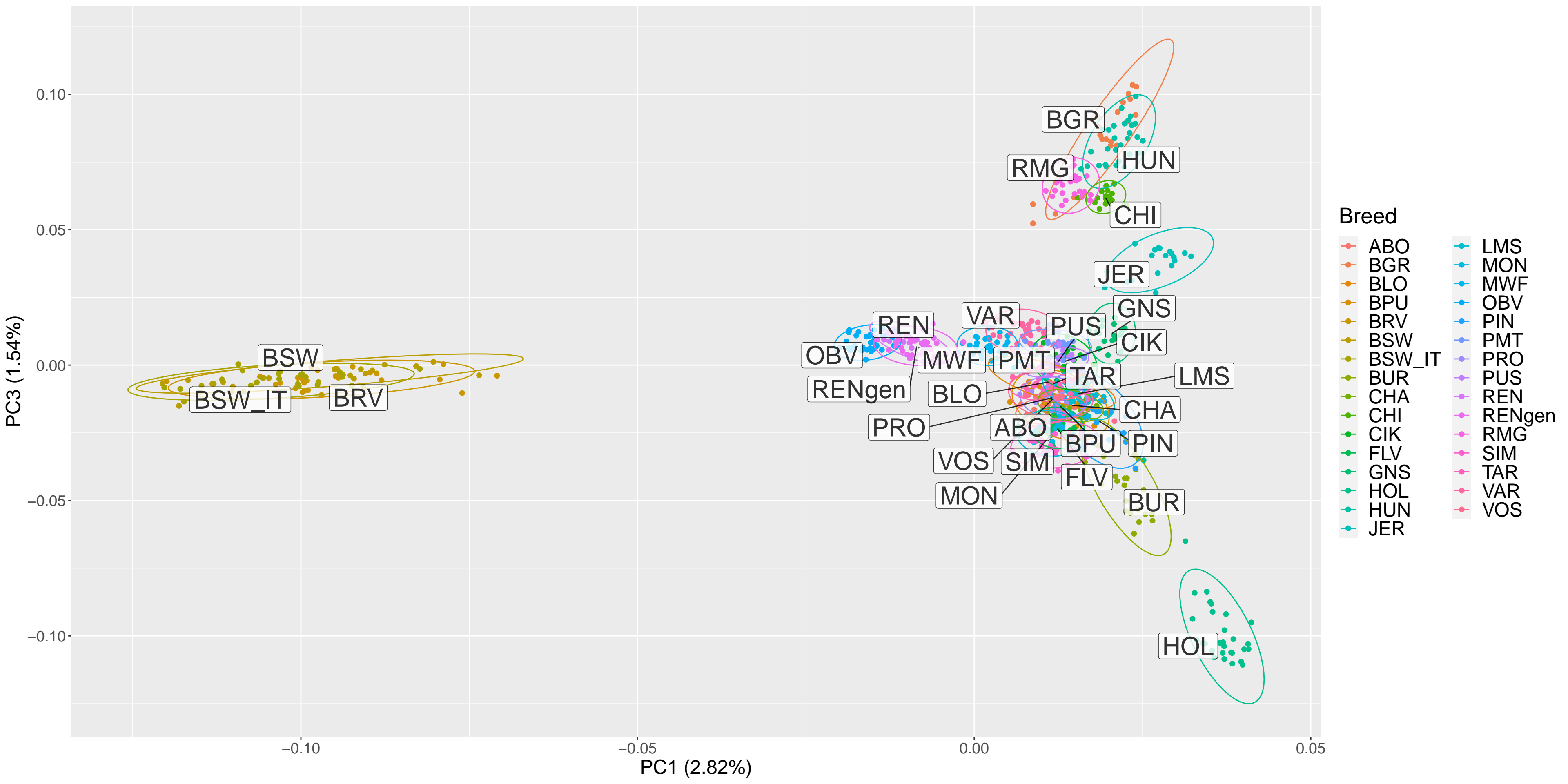

Supplement: Supplementary file 4 — Additional file 4: Figure S1. Principal component analysis (PC1 vs. PC3). The variance explained by each component is given as percentage in brackets. See Additional file 1: Table S1 for breed abbreviations. [file 12711_2024_900_MOESM4_ESM.pdf]

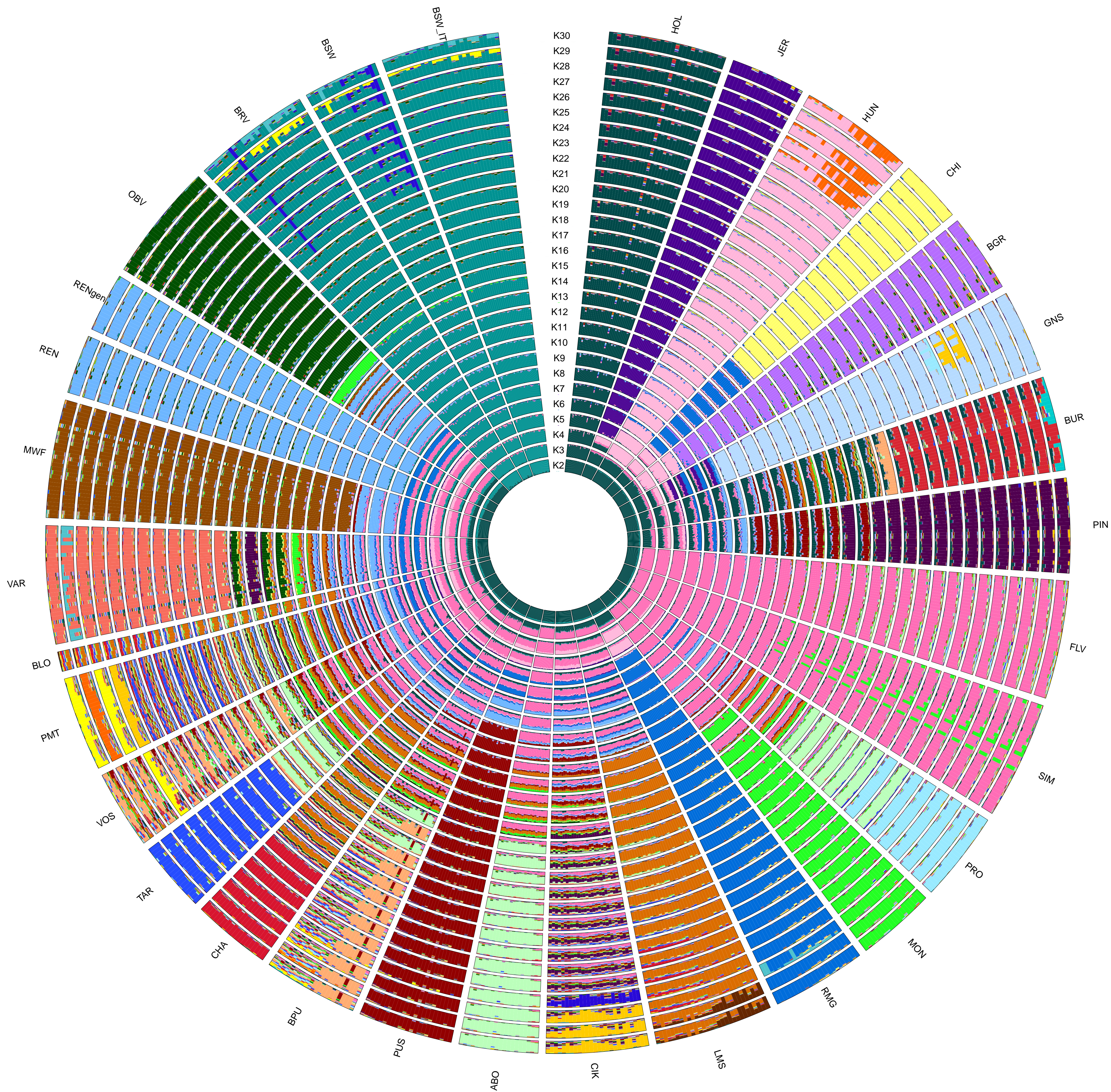

Supplement: Supplementary file 5 — Additional file 5: Figure S2. Circular plot of Admixture results for K from 2 to 30 displayed using a colour-blind friendly palette. Breeds are ordered accordi g to K = 2 values. See Additional file 1: Table S1 for breed abbreviations. [file 12711_2024_900_MOESM5_ESM.pdf]

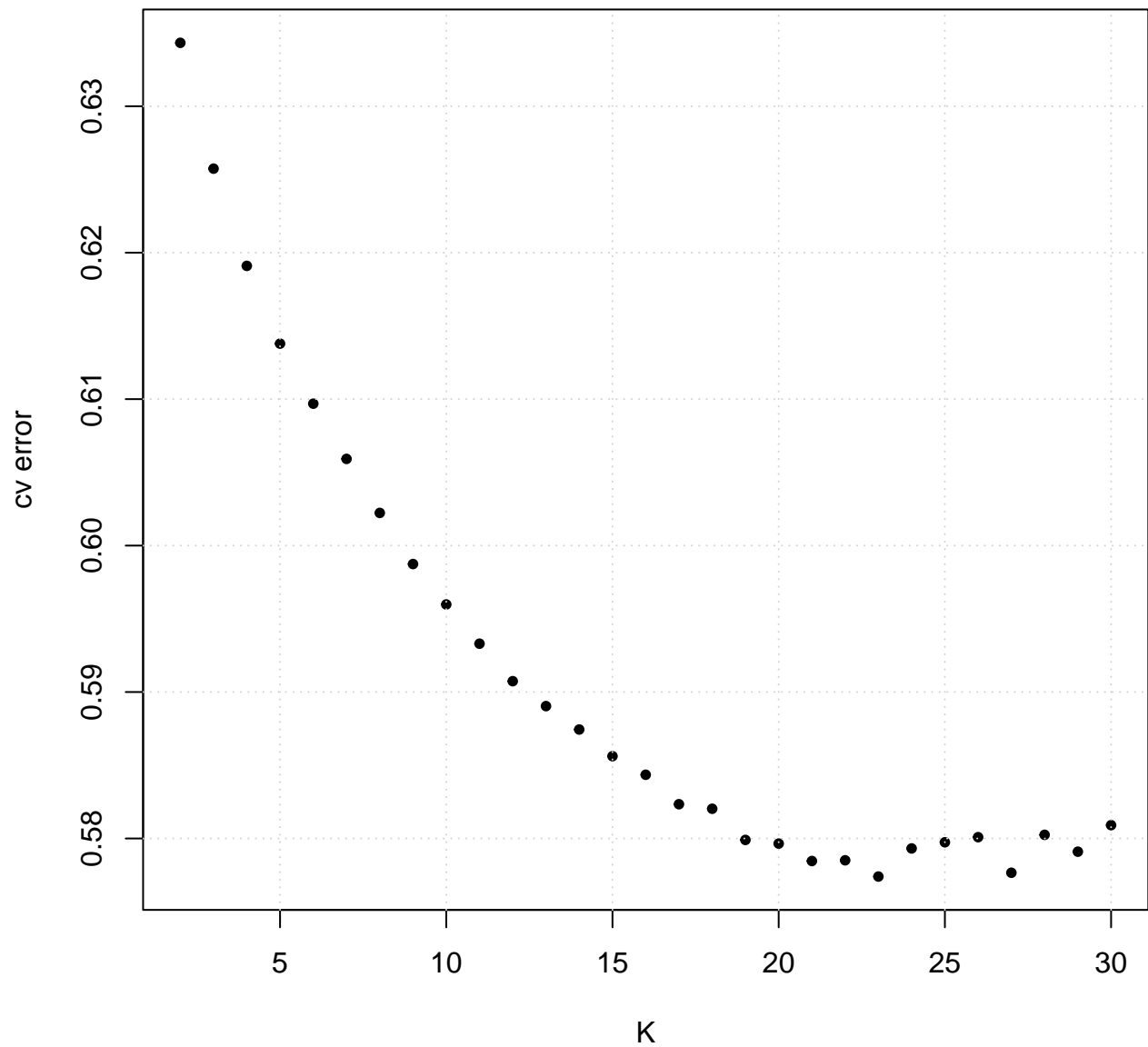

Supplement: Supplementary file 6 — Additional file 6: Figure S3. Cross-validation error values for K values from 2 to 30. [file 12711_2024_900_MOESM6_ESM.pdf]

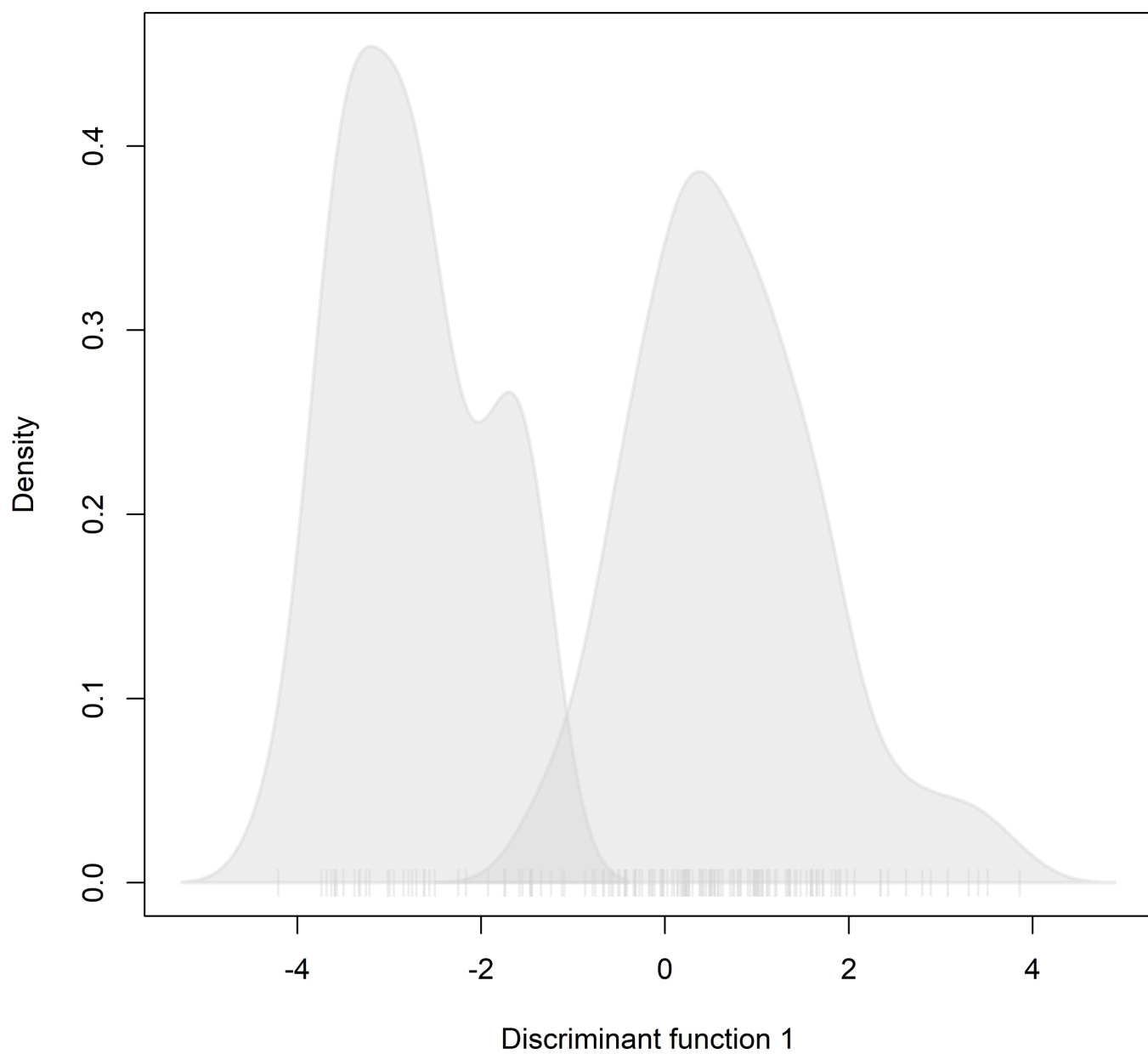

Supplement: Supplementary file 7 — Additional file 7: Figure S4. Discriminant analysis of principal component. Discriminant function 1 computed for the Rendena2000 and Rendena2018 populations. [file 12711_2024_900_MOESM7_ESM.pdf]

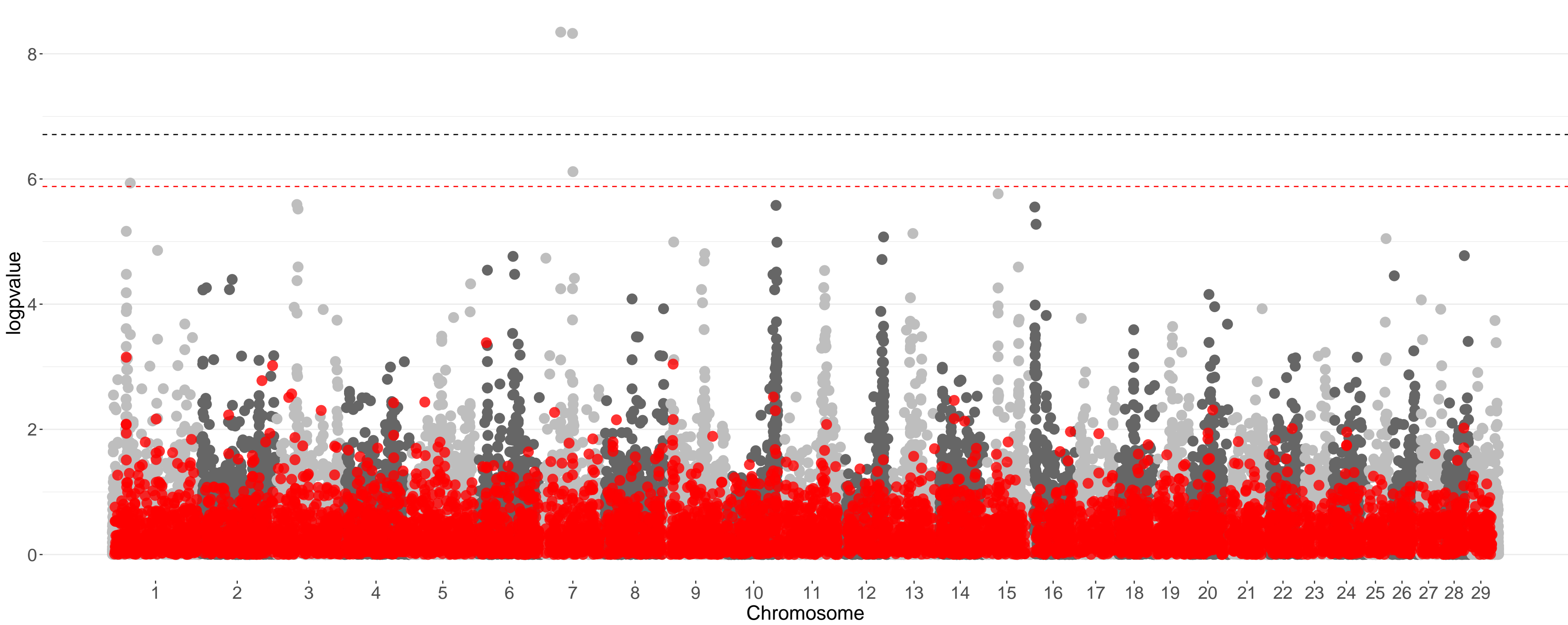

Supplement: Supplementary file 9 — Additional file 9: Figure S5. iHS results for the Rendena breed. In grey the analysis performed with all SNPs, in red the analysis performed using only SNPs for which the information on the ancestral allele was available. The dotted red and grey lines represent the significance threshold for iHS with and without ancestral allele information, respectively. [file 12711_2024_900_MOESM9_ESM.pdf]
