# Supplementary material for: Genetic legacy and adaptive signatures: investigating the history, diversity, and selection signatures in Rendena cattle resilient to eighteenth century rinderpest epidemics
Source: Genet Sel Evol. 2024 May 2;56:32. doi: 10.1186/s12711-024-00900-y (PMC11064358; doi:10.1186/s12711-024-00900-y)
Supplement: Supplementary file 8 — Additional file 8: Table S4. For each bovine chromosome, are listed the genes annotated for the most represented SNPs in the ROH analysis for the RENgen individuals. [file 12711_2024_900_MOESM8_ESM.docx]

| **Bovine chromosome** | **Genes annotated for SNPs identified in the ROH analysis** |
| --- | --- |
| *3* | *RNPC3* |
| *4* | *INSIG1, CNPY1, RBM33, ENSBTAG00000054159.*  *ENSBTAG00000052920, ENSBTAG00000052934, LMBR1, ENSBTAG00000049765, UBE3C, DNAJB6, HGF* |
| *5* | *ETNK1, C2CD5, ST8SIA1, ABCC9, LDHB, GYS2, RECQL, PYROXD1, SLCO1A2, SLCO1B3* |
| *6* | *FAM13A, HERC3, HERC6, ABCG2, PKD2, SPP1, MEPE, IBSP, LAP3, FAM184B, NCAPG, LCORL, SLIT2, KCTD8, YIPF7, GUF1, GNPDA2, GABRG1, GABRA2* |
| *10* | *RGS6, DPF3, DCAF4, ZFYVE1, PSEN1, PAPLN, NUMB, HEATR4, ACOT2, ENSBTAG00000009788, DNAL1, MIDEAS, PTGR2, ZNF410, FAM161B, ENTPD5, ALDH6A1, LIN52, VSX2, SYNDIG1L, LTBP2, ENSBTAG00000055057, YLPM1, PROX2, DLST, MLH3, ZC2HC1C, NEK9, TMED10, JDP2, FLVCR2, ENSBTAG00000011985, TTLL5, TGFB3, IFT43, GPATCH2L, ESRRB, ANGEL1, LRRC74A, TMEM63C, POMT2, TMED8, VIPAS39, SPTLC2, ALKBH1, SNW1, ADCK1, NRXN3, ENSBTAG00000049446* |
| *12* | *GPC6, ENSBTAG00000054671, ENSBTAG00000051263. DCT, TGDS* |
| *13* | *MPP7* |
| *16* | *NFASC* |
| *22* | *TRAK1, VIPR1, CCDC13, ACKR2, GASK1A, POMGNT2, ENSBTAG00000053758, SNRK, TOPAZ1, ZKSCAN7, ENSBTAG00000019479, IRAK2, VHL, FANCD2, IL17RC, ARPC4, TADA3, CPNE9* |
| *25* | *XYLT1* |
