# Supplementary material for: Genetic legacy and adaptive signatures: investigating the history, diversity, and selection signatures in Rendena cattle resilient to eighteenth century rinderpest epidemics
Source: Genet Sel Evol. 2024 May 2;56:32. doi: 10.1186/s12711-024-00900-y (PMC11064358; doi:10.1186/s12711-024-00900-y)
Supplement: Supplementary file 10 — Additional file 10: Table S5. Chromosome, marker, genomic position, XP-EHH values in the top 1% scored in the comparison between RENgen and Original Braunvieh data, RENgen and Brown Swiss cluster and annotated genes. In bold are markers scoring a value of XP-EHH above the threshold in both comparisons. [file 12711_2024_900_MOESM10_ESM.docx]

| **CHR** | **SNP** | **POSITION** | **XPEHH RENgen-OBV** | **XPEHH**  **RENgen-BSW** | **Annotated Genes** |
| --- | --- | --- | --- | --- | --- |
| 1 | **BTA-91456-no-rs** | **34,021,093** | **3.05** | **2.54** | *TMPRSS15,*  *CADM2* |
|  | BTB-00016647 | 33342088 | 2.921 | - |  |
|  | BTB-00930215 | 34424718 | - | 3.23 |  |
|  | BTB-01965833 | 34602877 | - | 2.30 |  |
|  | Hapmap49364-BTA-89353 | 34500276 | - | 2.69 |  |
|  | Hapmap53234-rs29020933 | 101090522 | 2.78 | - |  |
|  | Hapmap55373-rs29027574 | 34360152 | - | 2.67 |  |
| 2 | **BTB-01929922** | **8184346** | **2.98** | **2.57** |  |
|  | **Hapmap51331-BTA-85692** | **8268876** | **2.552** | **2.24** |  |
| 3 | BTA-122016-no-rs | 38681860 | - | 2.24 | *ENSBTAG00000015180* |
|  | BTA-67833-no-rs | 42687224 | - | 2.44 |  |
|  | **BTB-01405471** | **40255612** | **2.56** | **2.74** |  |
|  | **BTB-01537126** | **47073791** | **2.82** | **2.27** |  |
|  | **Hapmap38207-BTA-19427** | **46590052** | **3.26** | **2.48** |  |
|  | **Hapmap50814-BTA-89905** | **38634859** | **2.59** | **2.32** |  |
|  | Hapmap51027-BTA-67630 | 39699270 | - | 2.86 |  |
|  | Hapmap60279-rs29011976 | 38984858 | - | 2.41 |  |
| 7 | Hapmap35191-BES11_Contig367_1030 | 53261474 | - | 2.27 |  |
| 8 | BTA-81825-no-rs | 73015373 | - | 2.32 | *RCL1* |
|  | **BTB-00357617** | **72930313** | **2.79** | **2.26** |  |
|  | Hapmap55166-rs29016846 | 39584890 | - | 2.23 |  |
| 9 | BTB-00932823 | **16000040** | **3.07** | **2.69** |  |
|  | BTB-01407900 | 15941765 | 3.15 | - |  |
|  | **BTB-01874104** | **16596500** | **2.63** | **2.89** |  |
|  | **BTB-02052278** | **16562830** | **2.88** | **2.86** |  |
|  | **Hapmap25309-BTA-153368** | **16629763** | **3.14** | **2.63** |  |
|  | **Hapmap49346-BTA-85236** | **16029519** | **2.71** | **2.56** |  |
| 10 | BTA-79342-no-rs | 88380327 | 2.34 | - | *TMEM63C* |
| 11 | Hapmap52826-rs29011405 | 6776731 | - | 2.53 | *ENSBTAG00000043317* |
| 14 | **BTA-34248-no-rs** | **21787017** | **2.80** | **2.37** |  |
| 15 | BTA-36547-no-rs | 36242730 | - | 2.62 | *SOX6* |
|  | BTB-01559194 | 36411602 | - | 2.41 |  |
|  | **BTB-01559217** | **36378628** | **3.18** | **3.08** |  |
|  | **BTB-02055779** | **36281231** | **2.83** | **3.36** |  |
|  | Hapmap43561-BTA-36544 | 36219316 | - | 2.61 |  |
| 16 | **BTB-01567754** | **41535754** | **3.82** | **2.27** | *VPS13D,*  *TNFRSF1B* |
| 17 | Hapmap34713-BES10_Contig634_914 | 18798920 | - | 2.93 |  |
| 18 | Hapmap44357-BTA-24055 | 31620559 | - | 2.23 |  |
| 20 | BTA-50529-no-rs | 37309348 | 2.79 | - | *WDR70,*  *CPLANE1,*  *NIPBL* |
| 23 | Hapmap42254-BTA-56665 | 44387829 | - | 2.34 | *HIVEP1* |
